# Rapid Nasal Breathing as a Biometric Trigger: High‐Accuracy Electroencephalogram‐Based Authentication for Clinical Applications

**DOI:** 10.1111/crj.70148

**Published:** 2026-01-23

**Authors:** Cai Chen, Xianghong Kong, Danyang Lv, Xiangwei Meng, Chongxuan Tian, Zhi Li, Fengxia Wu, Ningling Zhang, Dedong Ma

**Affiliations:** ^1^ Shandong Institute of Advanced Technology Chinese Academy of Sciences Jinan China; ^2^ Department of Cosmetic Surgery Chengdu Badachu Medical Aesthetics Hospital Chengdu China; ^3^ Biomedical Engineering Institute, School of Control Science and Engineering Shandong University Jinan China; ^4^ School of Basic Medical Sciences Shandong University Jinan China; ^5^ Department of Pulmonary and Critical Care Medicine Qilu Hospital Shandong University Jinan China; ^6^ School of Medicine Xizang University Lhasa Xizang Autonomous Region China

**Keywords:** biometric recognition, clinical authentication, deep learning, electroencephalography, respiratory patterns

## Abstract

**Background:**

Traditional biometric systems are vulnerable to forgery, highlighting the need for secure alternatives. Electroencephalography (EEG) offers inherent advantages in liveness detection and antispoofing but typically requires external stimuli. We propose a novel paradigm leveraging intrinsic respiratory‐evoked EEG signals for identity authentication, with potential applications in clinical settings where unobtrusive monitoring is critical.

**Methods:**

We developed a 64‐channel EEG acquisition system with synchronized respiratory event monitoring. Thirteen healthy volunteers performed four breathing patterns: oral, nasal, slow nasal, and rapid nasal breathing. A hybrid deep learning model was designed to optimize spatial–temporal feature extraction from EEG signals.

**Results:**

The model achieved 98.3% accuracy in identity recognition using rapid nasal breathing‐evoked EEG, outperforming traditional biometric methods. Nasal breathing patterns consistently yielded higher accuracy than oral breathing, with rapid nasal breathing showing the strongest discriminative power.

**Conclusions:**

Respiratory‐evoked EEG signals provide a viable, noninvasive biometric identifier. The high accuracy of rapid nasal breathing opens avenues for clinical integration, such as continuous patient authentication in respiratory monitoring devices or secure access to electronic health records.

## Graphical abstract

Our study demonstrates rapid nasal breathing‐evoked EEG signals enable high‐accuracy (98.3%) biometric authentication via a hybrid ResNet‐Swin Transformer model. This noninvasive approach eliminates external stimuli, offering secure patient identification for critical respiratory care settings, such as ventilator access and continuous monitoring.

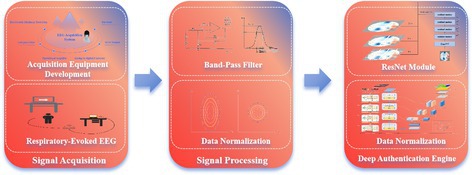

## 1 Introduction

Biometric identification systems leverage biosensors to authenticate individuals based on unique biological characteristics, encompassing both physiological attributes (e.g., fingerprints [1], facial features [2], and deoxyribonucleic acid profiles [3]) and behavioral traits (e.g., voice patterns [4], handwriting signatures [5], and gait characteristics [6]). From a theoretical perspective, any biometric feature could be applied to identity recognition provided it satisfies four fundamental criteria: universality, uniqueness, stability, and collectability.

We conducted a literature review by using the keywords “biometric recognition, electroencephalogram (EEG) signals, and respiration” in the PubMed database for the search. Although EEG‐based biometric identification has been extensively explored using cognitive‐task paradigms such as motor imagery, visual evoked potentials, and resting‐state analysis, these methods encounter persistent challenges in real‐world clinical environments. Their performance is often affected by variability in mental state, environmental conditions, and the inability to integrate identity verification with simultaneous health monitoring. Nevertheless, contemporary biometric modalities remain susceptible to counterfeiting and coercive exploitation. Published research confirms that synthetic gelatin replicas can reliably deceive fingerprint recognition systems [7], and Schultz et al. have documented a three‐dimensional fingerprint replication method capable of accurately recording dermatoglyphic minutiae [7]. While countermeasures such as iris recognition enhance liveness detection capabilities, their efficacy against sophisticated adversarial attacks remains constrained [8]. Consequently, in high‐security domains including military and defense infrastructure, conventional biometric traits retain vulnerabilities to compromise. Identity verification based on EEG data has therefore attracted substantial interest, facilitated by advancements in electrode sensor technology [9].

EEG constitutes an electrical phenomenon observable on the cerebral cortex or scalp surface, resulting from ionic currents generated during neuronal information transmission [10]. The neurological system inherently produces autonomous signals while exhibiting adaptive neuroplastic modifications in response to environmental stimuli. EEG signals confer distinct advantages for biometric applications such as liveness dependency and nonreplicability. Signals originate exclusively from conscious, cooperative subjects; coerced individuals exhibit quantifiably distinct electrophysiological patterns [11]. Intersubject neurophysiological variability and intrasubject state‐dependent dynamics preclude signal duplication [12]. These properties—concealability, unstealability, unforgeability, noncoercibility, and liveness dependence—position EEG as a novel biometric modality capable of overcoming limitations inherent to conventional systems.

Research on EEG‐based identity authentication has diversified significantly. Primary mechanisms exploit event‐related potential (ERP) modifications evoked by external stimuli, categorized as motor imagery, visual evoked potentials (VEPs), and P300 potentials. Motor imagery studies have shown that cortical activation during kinesthetic imagination elicits detectable EEG alterations [11, 12], achieving up to 98.97% recognition accuracy with support vector machines on multimodal features [13] or 94.13% accuracy with channel‐optimized autoregressive coefficients [14]. Visual stimulus paradigms such as steady‐state oscillations and auditory evoked potentials have yielded accuracies above 96% [15, 16]. Cognitive processing via oddball paradigms has also demonstrated potential, with consumer‐grade devices reaching 72% [17] and laboratory setups achieving 83.1% [18]. However, these approaches predominantly rely on external stimulus presentation systems—visual displays, auditory stimulators—which limit portability and confine application to controlled laboratory environments.

Respiratory–EEG interactions are a well‐documented neurophysiological mechanism explored in various contexts. Cortical activity changes during respiratory challenges have been observed in apnea tests (alpha frequency reduction) [19], neonatal apnea (EEG suppression and decreased amplitude/frequency) [20], and voluntary respiration control scenarios [21]. Respiration rhythm provides a temporal modulation component to ongoing cortical activity [22] and influences neuro–cognitive circuitry, with effects on stress reduction and cognition via brain stem and subcortical pathways [23]. Traditional authentication fails in critical care scenarios—intubated patients cannot provide fingerprints and delirium compromises password recall. Respiratory–EEG biometrics enable continuous, noninvasive authentication integrated into therapeutic monitoring: secure ventilator access for COPD/OSA home noninvasive ventilation (NIV) therapy, automated identity verification during polysomnography, and tamper‐proof safety locks in opioid‐induced respiratory depression monitors. Volitional breathing modulates EEG spectral properties [24] and rapid breathing engages occipital regions [25]. Existing EEG biometrics rely on externally evoked potentials and peripheral hardware [13, 16], limiting portability and wearability.

To address these gaps, we propose a novel identity recognition method based on intrinsic physiological signals—specifically rapid–nasal–respiration–evoked EEG—which eliminates dependence on external apparatus while achieving high accuracy. This dual‐functional system delivers secure identity authentication and real‐time detection of respiratory anomalies. Key contributions include (1) introduction of a controlled physiological trigger in EEG biometrics for improved repeatability; (2) demonstration of integrated clinical monitoring and authentication in one framework; and (3) deployment of a custom 64‐channel acquisition platform and hybrid deep learning pipeline optimized for spatial–temporal feature analysis.

Our assumptions include the reproducibility of respiratory patterns under standardized conditions and accessibility of specialized hardware; limitations include the relatively small sample size and current dependence on high‐density EEG instrumentation.

The remainder of this paper is organized as follows: Section 1 (Related Research) presents a comparative literature review covering conventional EEG biometric methods, physiological–stimulus approaches, and respiratory neurophysiology studies. Section 2 (Materials and Methods) details participant recruitment, experimental design, and signal processing. Section 3 (Results) reports identity authentication accuracy and respiratory anomaly detection performance. Section 4 (Discussion) explores clinical integration, ethical considerations, and technical limitations. Section 5 concludes with implications and future work.

## 2 Methodology

### 2.1 System Architecture Overview

**FIGURE 1:**
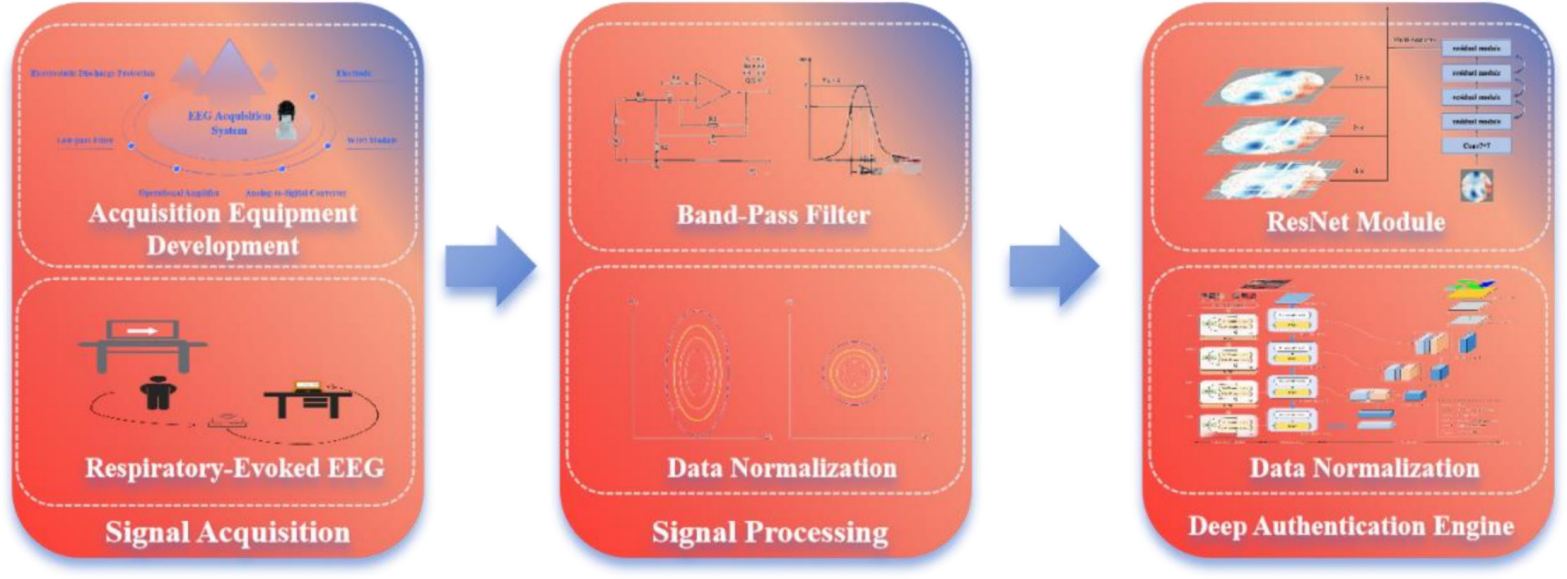
Three core components of the proposed respiratory–evoked EEG biometric system, showing signal acquisition with a custom EEG device for capturing respiratory‐induced brain activity, signal processing including band‐pass filtering and normalization to reduce noise and intersubject variability, and a deep authentication engine that applies a ResNet‐based model for spatial–temporal feature extraction and identity verification.

The proposed identification framework integrates respiratory–brain interaction analysis with multimodal biometric verification. As illustrated in Figure 1, the system comprises three core modules.

1 Neurophysiological Signal Acquisition

A wearable EEG system (64‐channel, 500‐Hz sampling rate) was employed to capture high‐fidelity electroencephalographic signals during controlled breathing paradigms.

2 Signal Processing Pipeline

Raw EEG data were preprocessed by excluding rest intervals and constructing spatiotemporal matrices with 0.1–30‐Hz band‐pass filtering. Channel‐wise *z*‐score normalization standardized EEG amplitudes across subjects. Four respiratory classes (mouth/nose breathing, slow/fast nasal patterns) were labeled to establish supervised classification frameworks.

3 Deep Authentication Engine

The features extracted by ResNet were taken as input and the hierarchical structure of Swin Transformer was used to gradually increase the level of abstraction of the features to capture higher levels of context information.

### 2.2 EEG Acquisition Amplifier

**FIGURE 2:**
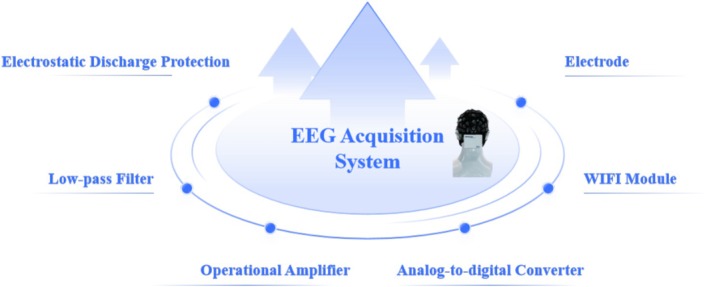
EEG acquisition amplifier, illustrating the custom‐designed EEG acquisition system comprising electrostatic discharge protection, low‐pass filtering to remove unwanted signal components, high‐gain operational amplifiers, an analog‐to‐digital converter for precise signal digitization, a flexible electrode array for scalp signal collection, and a Wi‐Fi module for wireless data transmission.

This study presents a modular EEG acquisition amplifier (Figure 2). The neurophysiological sensor array employs a 64‐channel flexible EEG cap with Ag/AgCl electrodes spaced according to the 10–20 international system, ensuring spatial resolution of 2.5 cm^2^ per channel. The EEG signal acquisition system is meticulously engineered to deliver exceptional performance, incorporating an ultrahigh input impedance of 1GΩ, an outstanding common‐mode rejection ratio (CMRR) exceeding 110 dB, and an extraordinarily low input noise floor below 0.4 μVrms. This configuration ensures robust signal integrity even in high‐interference environments. The system achieves a single‐channel sampling rate of up to 2000 samples per second (SPS), enabling high‐fidelity capture of fine‐grained neural dynamics. Notably, under typical operational loads, it maintains a battery life of up to 4.5 h while adhering to a lightweight design of just 130 g (inclusive of the amplifier and battery), ensuring prolonged user comfort during extended use.

**FIGURE 3:**
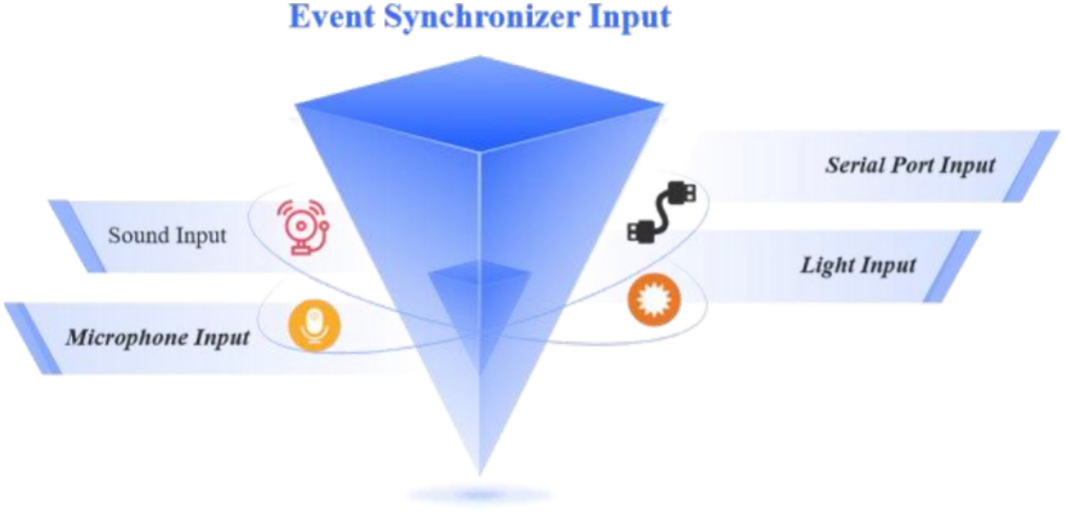
Event synchronizer input, showing the multiple channels used to trigger and align data acquisition events, including sound input, microphone input for voice‐based signals, serial port input for device communication, and light input for optical stimulus synchronization.

ERPs are the electrophysiological responses elicited by the brain in response to specific events, such as visual, auditory, or tactile stimuli [6]. In EEG research, precise temporal marking of these events is crucial when recording ERPs. The event synchronizer plays a vital role in ensuring that the timing of stimulus event recordings in the experiment aligns accurately with the EEG recordings. In our system, the event synchronizer is integrated with both the stimulus presentation software and the EEG acquisition software. By continuously monitoring and inspecting the timing, any transmission delays are effectively eliminated. This ensures that the event recordings and EEG recordings are synchronized with remarkable precision, maintaining an error margin of less than 1 ms. The functional structure of the event synchronization system is depicted in Figure 3. The event synchronizer is designed to receive a variety of input signals, including light, audio, and microphone signals, as well as inputs from serial ports.

### 2.3 Signal Acquisition

This study enrolled 13 healthy adults who completed all experimental tasks under standardized conditions. Data were acquired using a 64‐channel high‐density EEG system with a 500‐Hz sampling rate, optimized to capture respiratory‐evoked cortical potentials [25, 26]. Participants performed four distinct breathing paradigms: oral breathing, nasal breathing, slow‐paced nasal breathing, and rapid‐paced nasal breathing. Each 30‐s task block was separated by 10‐s inter‐trial intervals to mitigate fatigue effects. We collected the evoked EEG signals of 13 individuals under 4 different breathing patterns, with each pattern repeated five times. To ensure protocol compliance and data consistency, participants underwent comprehensive training sessions prior to the experiment. Additionally, a pre‐experiment health screening questionnaire was administered to exclude individuals with neurological, psychiatric, or respiratory conditions that could confound the results.

### 2.4 Data Processing

Rest intervals were excluded during analysis to isolate task‐specific neural activity. The 64‐channel biosensor data were structured into a two‐dimensional matrix (64 electrodes × temporal sampling points) with synchronized timestamps. Subsequently, band‐pass filtering (0.1–30 Hz) was applied to eliminate low‐frequency drift and high‐frequency noise while preserving ERPs. To mitigate intersubject variability in EEG signal amplitude, channel‐wise min–max normalization was performed:

(1) $$x^{\prime }=\frac{x-\min\left(x\right)}{\max\left(x\right)-\min\left(x\right)}$$

where *x* and *x′* denote original and normalized values, respectively. This transformation standardizes all channels to a [0,1] range, enhancing cross‐participant comparability and subsequent model robustness.

Each 30‐s EEG segment (500‐Hz sampling) was partitioned into 64 temporal frames to capture dynamic neural patterns. Through systematic reorganization, a 64 × 64 spatiotemporal matrix was constructed with spatial dimension (electrode positions, horizontal axis) and temporal dimension (sequential signal frames, vertical axis). This representation preserves both spatial topography and temporal dynamics for deep learning applications. All epochs were labeled according to respiratory kinematics (1) mouth breathing, (2) nose breathing, (3) slow nasal breathing, and (4) rapid nasal breathing. These classifications establish supervised learning targets, enabling neural network recognition of respiratory patterns.

### 2.5 Model Evaluation

Accuracy is the fraction of samples correctly identified by the model out of the total number of samples. The calculation formula was shown in Equation (2).

(2) $$\text{Accuracy}=\frac{\mathrm{TP}+\mathrm{TN}}{\mathrm{TP}+\mathrm{FP}+\mathrm{FN}+\mathrm{TN}}$$

True positive (TP) indicates the true example, true negative (TN) indicates the true example, false positive (FP) indicates the false positive example, and false negative (FN) indicates the false negative example. Precision refers to the proportion of samples predicted by the model to be positive that are actually positive. The calculation formula was shown in Equation (3).

(3) $$\text{Precis}i\mathrm{on}=\frac{\mathrm{TP}}{\mathrm{TP}+\mathrm{FP}}$$

The recall rate is the proportion of the sample that is actually positive and is correctly predicted to be positive by the model, shown in Equation (4).

(4) $$\text{Recall}=\frac{\mathrm{TP}}{\mathrm{TP}+\mathrm{FN}}$$

Specificity refers to the proportion of a sample that is actually a negative class and that is correctly predicted by the model to be negative.

(5) $$\text{Specificity}=\frac{\mathrm{TN}}{\mathrm{TN}+\mathrm{FP}}$$

The F1 score is a harmonic average of the accuracy and recall rates used to comprehensively evaluate the performance of the model. The calculation formula is shown in Equation (6).

(6) $$F1\;\text{Score}=2\times \frac{\text{Precision}\times \text{Recall}}{\text{Precision}+\text{Recall}}$$

### 2.6 Proposed Method—Multimodal Fusion Neural Network Architecture

In this study, the feature extraction power of the model is improved by integrating the benefits of ResNet with Swin Transformer. The detailed steps are outlined below:

#### 2.6.1 Initial Feature Extraction

**FIGURE 4:**
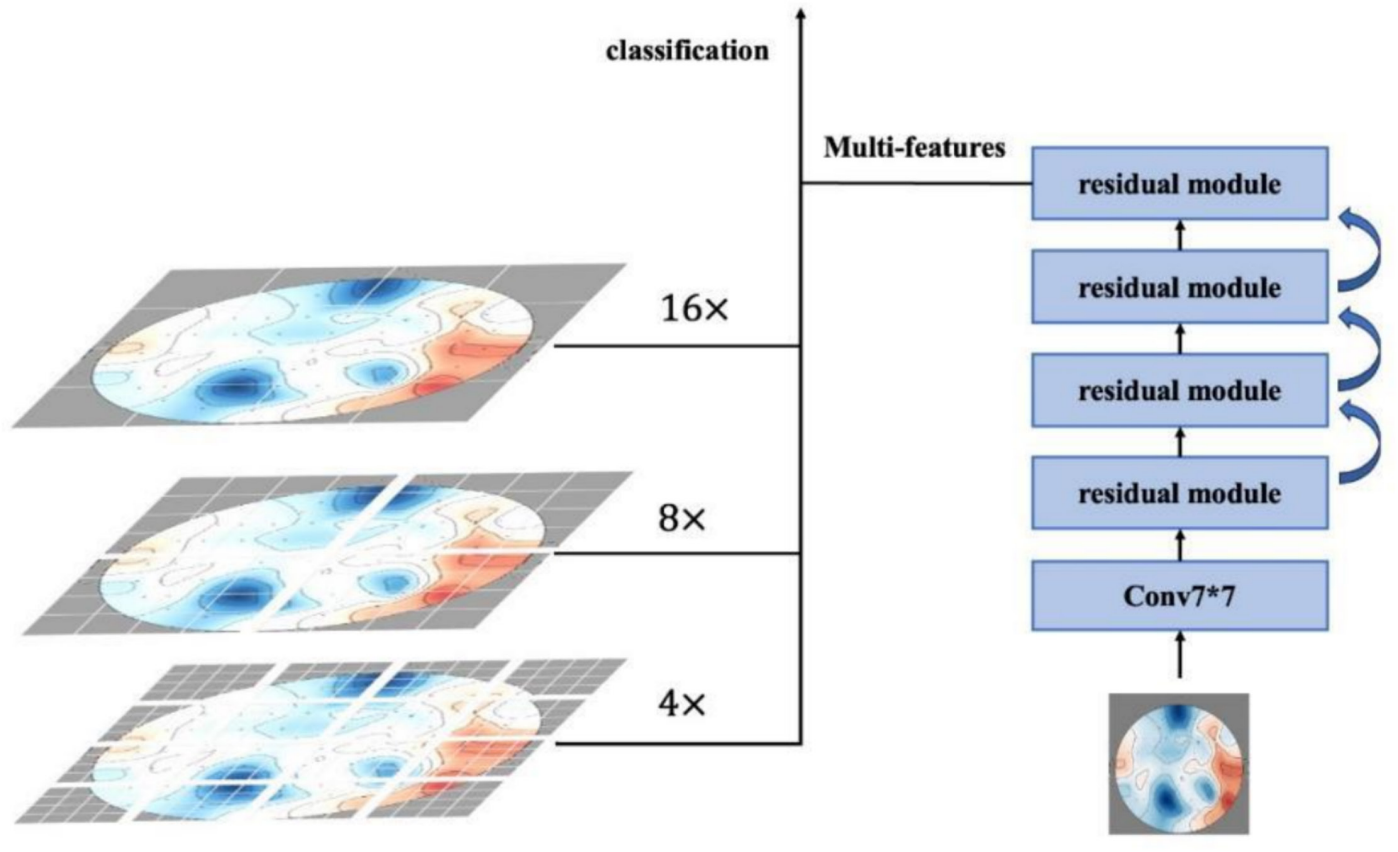
Multiscale feature extraction process of the ResNet module, where EEG spatial–temporal maps are processed at three resolutions (16×, 8×, 4× downsampling) to capture both coarse and fine spatial details. The resulting multiscale features are passed through a 7 × 7 convolution layer and successive residual modules to enhance representation before being integrated for classification.

ResNet‐50 functions as the primary feature extractor, leveraging its deep residual architecture to hierarchically capture localized image attributes through stacked convolutional layers, pooling operations, and residual blocks. This design ensures sensitivity to subtle spatial variations. In our implementation, we removed the final classification layer to preserve all preceding feature extraction capabilities and implemented multiscale processing at 16×, 8×, and 4× downsampling ratios via parallel residual pathways. And then, we integrated feature maps through 7 × 7 convolutional fusion, enhancing representational richness across spatial frequencies (Figure 4).

#### 2.6.2 Feature Fusion

Following the completion of the preceding two phases, we integrate the features extracted from the two models using a feature fusion algorithm. Specifically, we concatenate features from ResNet's final layer output and Swin Transformer to create a new feature vector. In addition, we investigate alternative feature fusion methods, such as weighted summation, to determine the best fusion strategy. The fused feature vector includes additional image information, such as local detail features and global context information. This fusion strategy enables the model to take advantage of the strengths of both models in order to perform better on a number of visual tasks.

#### 2.6.3 Fully Connected Layers

The fused features were sent into the Fully Connected Layers, which executed the final classification, regression, and other tasks. The fully connected layer maps high‐level features to the output space necessary for a specific job, completing the model's prediction process. In this study, we performed the classification task with a fully connected layer and employed the Softmax function as the output activation function.

#### 2.6.4 Model Structure

**FIGURE 5:**
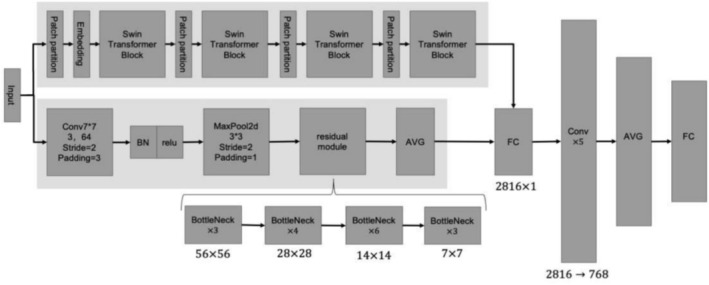
Multimodal fusion neural network architecture, combining a ResNet‐50 module for multiscale local feature extraction and a Swin Transformer module for global context modeling. Features from both branches are fused and passed through convolutional layers, average pooling, and fully connected layers to perform classification, enabling robust respiratory‐evoked EEG recognition.

This study introduces a novel multimodal fusion neural network architecture, combining the strengths of Swin Transformer and ResNet to enhance breath activity recognition performance. The network structure is illustrated in Figure 5.

#### 2.6.5 Training Strategy

To ensure stable convergence and optimal performance of our integrated model, we employed the following training procedures. (1) Pre‐trained Models: We leveraged pre‐trained ResNet and Swin Transformer models to enhance initial performance and accelerate convergence. Specifically, we utilized ResNet‐50 and Swin‐T models pre‐trained on ImageNet for initialization. (2) Regularization: To prevent overfitting, we dynamically adjusted the learning rate using regularization techniques like heavy decay throughout the training process. (3) Optimization: We improved model parameters by using the AdamW optimizer combined with learning rate scheduling algorithms such as Cosine Annealing. (4) Data Augmentation: We applied various data augmentation techniques, including random cropping, flipping, and color dithering, to enhance the model's robustness and generalization capabilities. Through these structural optimization techniques, we aimed to develop a model capable of capturing both local and global features, thereby improving performance across multiple visual tasks.

In summary, this study combines respiratory‐evoked EEG signal acquisition with deep learning‐based multiscale and multimodal feature extraction for biometric identification. EEG data were collected from 13 participants under four distinct breathing patterns, with each pattern repeated five times, using a custom 64‐channel system configured for high‐fidelity recording. Preprocessed signals were transformed into spatiotemporal feature matrices and analyzed using an integrated framework comprising ResNet‐50 and Swin Transformer architectures. Multiscale features from the ResNet module and global contextual features from the Swin Transformer were fused to enhance representation. Model optimization was performed using the Adam optimizer with cosine annealing learning rate scheduling, along with data augmentation to improve generalization. System performance was evaluated across accuracy, precision, recall, specificity, and F1‐score metrics for all breathing conditions.

## 3 Result

### 3.1 Oral vs. Nasal Respiration Comparison

**FIGURE 6:**
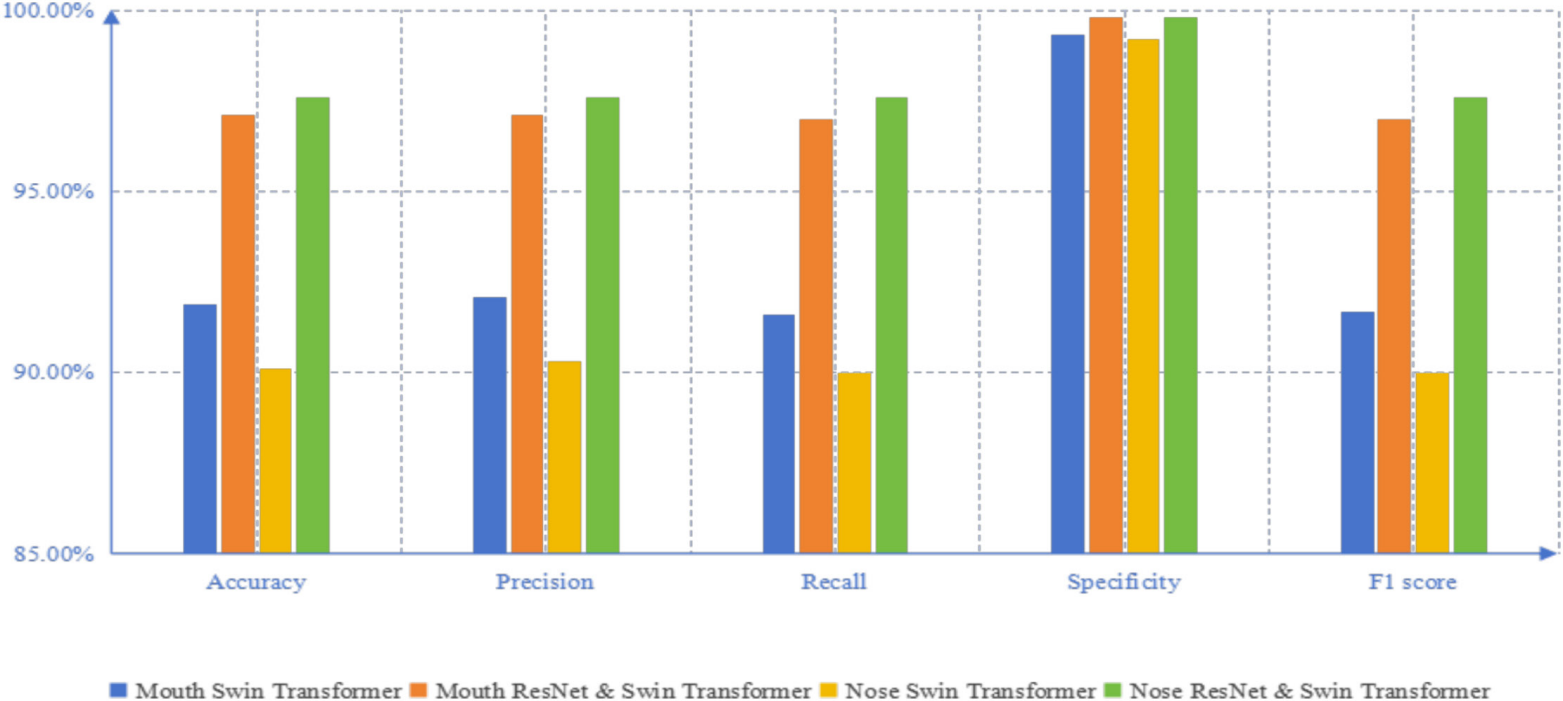
Performance comparison of oral and nasal breathing patterns in EEG‐based identity recognition, showing accuracy, precision, recall, specificity, and F1‐score for two classification models (Swin Transformer and ResNet & Swin Transformer). Results indicate that nasal breathing consistently achieves slightly higher performance than oral breathing, with the ResNet & Swin Transformer model providing the best overall results across all metrics.

Through EEG analysis of respiratory patterns, we observe distinct performance differences between oral and nasal breathing using Swin Transformer as baseline model in Table 1 and Figure 6. Oral breathing achieved 91.9% accuracy (precision = 92.1%, recall = 91.6%, specificity = 99.3%, and F1 = 0.917), while nasal breathing attained 90.1% accuracy (precision = 90.3%, recall = 90.0%, specificity = 99.2%, and F1 = 0.900). Our integrated ResNet & Swin Transformer model demonstrated superior performance, which is that oral breathing reached 97.1% accuracy (precision = 97.1%, recall = 97.0%, specificity = 99.8%, and F1 = 0.970) and nasal breathing achieved 97.6% accuracy (precision = 97.6%, recall = 97.6%, specificity = 99.8%, and F1 = 0.976). Crucially, though standalone Swin Transformer showed marginal oral breathing advantage (91.9% vs. 90.1% accuracy), nasal respiration exhibited more balanced metric performance. The hybrid model enhanced nasal breathing recognition by 7.5 percentage points compared to baseline, ultimately outperforming oral breathing by 0.5 percentage points in accuracy.

**TABLE 1:** Identification based on EEG signal evoked by mouth breathing and nose breathing.

Breathing patterns	Model	Accuracy	Precision	Recall	Specificity	F1 score
Mouth	Swin Transformer	91.9%	92.1%	91.6%	99.3%	0.917
Mouth	ResNet & Swin Transformer	97.1%	97.1%	97.0%	99.8%	0.970
Nose	Swin Transformer	90.1%	90.3%	90.0%	99.2%	0.900
Nose	ResNet & Swin Transformer	97.6%	97.6%	97.6%	99.8%	0.976

### 3.2 Nasal Respiratory Flow Rate Analysis

Classification performance across nasal breathing intensities reveals critical variations under Swin Transformer baseline as shown in Table 2. Rapid breathing registered 95.2% accuracy (precision = 95.1%, recall = 95.0%, specificity = 99.6%, F1 = 0.950), slow breathing 95.9% (precision = 96.1%, recall = 95.7%, specificity = 99.7%, F1 = 0.956), and normal breathing 90.1%. The ResNet & Swin Transformer fusion model significantly improved recognition. Rapid breathing increased to 98.3% accuracy (precision = 98.3%, recall = 98.3%, specificity = 99.9%, F1 = 0.983), slow breathing to 97.8% (precision = 97.8%, recall = 97.7%, specificity = 99.8%, F1 = 0.977), and normal breathing to 97.6%. This represents gains of +3.1, +1.9, and +7.5 percentage points respectively. Notably, slow breathing achieved the highest baseline accuracy (95.9%), suggesting its distinctive EEG signatures enhance detectability, while the fused model showed peak performance in rapid breathing recognition (98.3%).

**TABLE 2:** Classification performance of different nasal respiratory flow rates.

Nasal breathing patterns	Model	Accuracy	Precision	Recall	Specificity	F1 score
Breathe fast	Swin Transformer	95.2%	95.1%	95.0%	99.6%	0.950
Breathe fast	ResNet & Swin Transformer	98.3%	98.3%	98.3%	99.9%	0.983
Breathe slowly	Swin Transformer	95.9%	96.1%	95.7%	99.7%	0.956
Breathe slowly	ResNet & Swin Transformer	97.8%	97.8%	97.7%	99.8%	0.977

### 3.3 Model‐Wise Respiratory Classification

**FIGURE 7:**
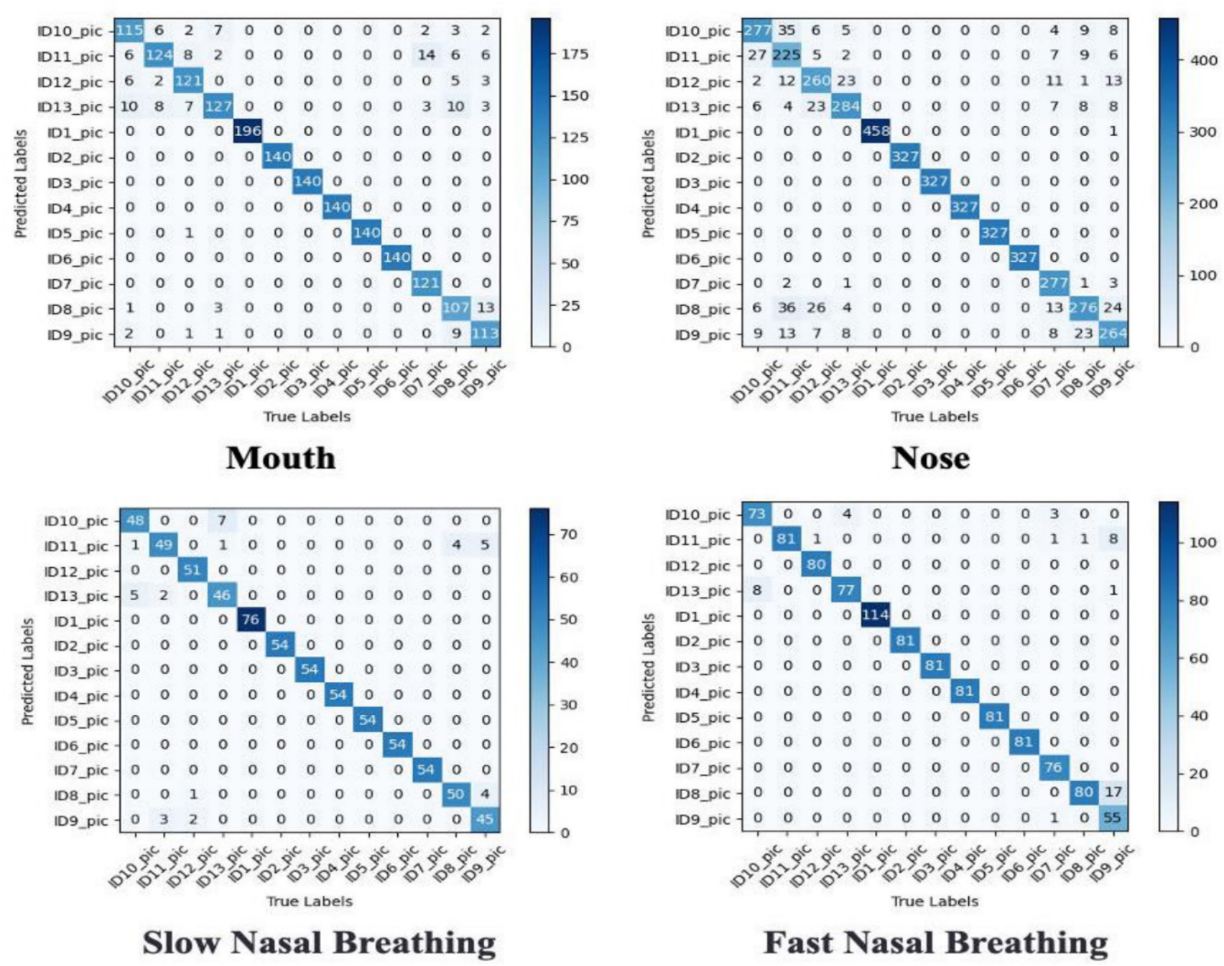
Confusion matrix for Swin Transformer.

**FIGURE 8:**
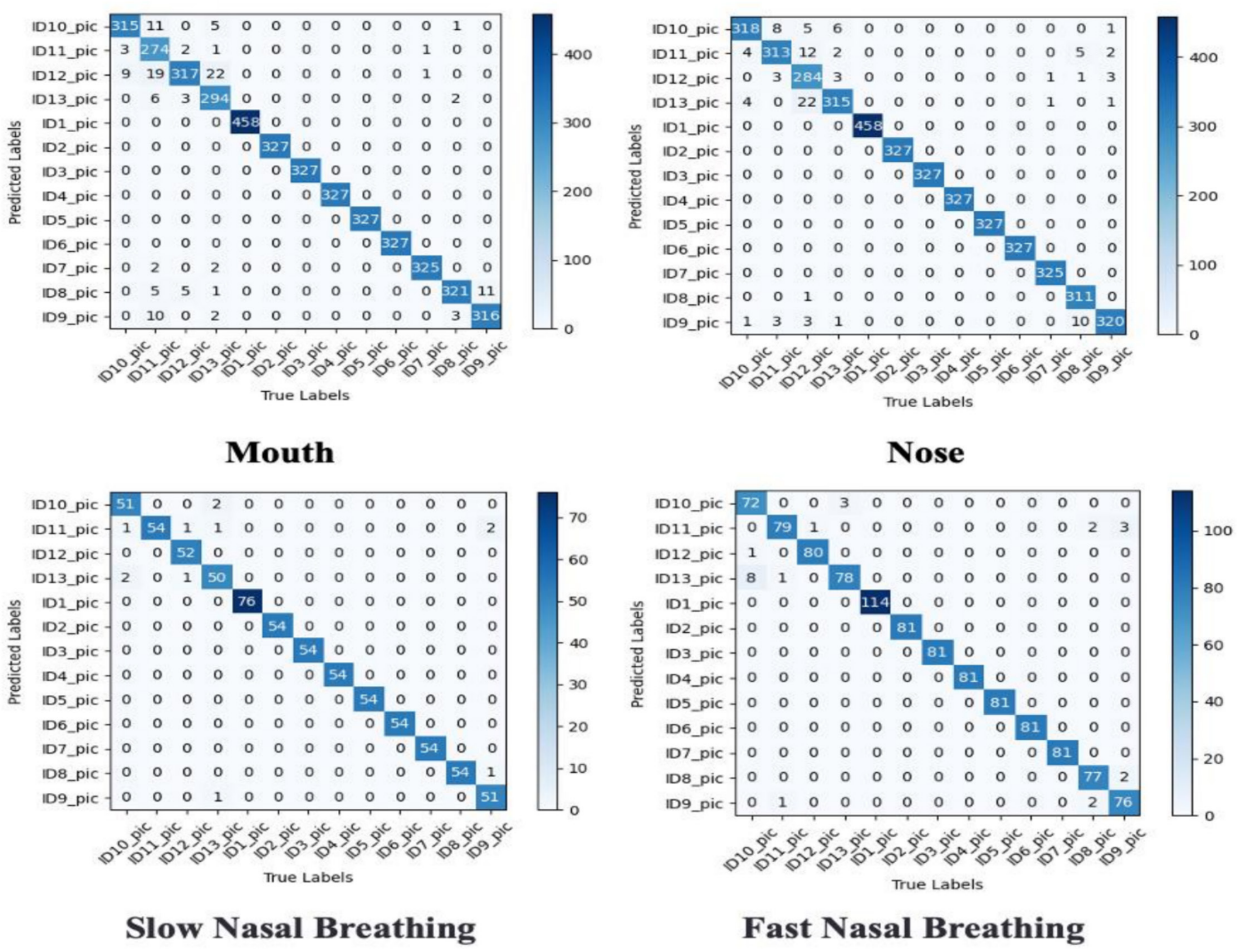
Confusion matrix for ResNet & Swin Transformer.

Swin Transformer demonstrates these characteristics in Figure 7. Oral breathing classifications remain predominantly correct, but confusion occurs between nasal types—approximately 12.3% of normal nasal trials misclassified as slow nasal breathing, while 8.7% of rapid nasal trials confused with normal breathing. Figure 8 illustrates ResNet & Swin Transformer's superior performance: Mouth breathing classification accuracy improves substantially, while nasal breathing confusion decreases markedly—normal vs. slow misclassification drops to approximately 3.3% with enhanced rapid‐normal differentiation. These comparative results verify that integrating ResNet's multiscale feature extraction with Swin Transformer significantly improves recognition of complex breathing patterns.

## 4 Discussion

This study establishes nasal breathing as a superior biometric modality compared to oral respiration, with peak accuracy (98.3%) achieved during rapid nasal breathing. This is related to the differences in electroencephalogram (EEG) signals induced by nasal breathing and mouth breathing.

### 4.1 Neurophysiological Basis of Nasal vs. Oral Breathing

The differences in electroencephalogram (EEG) signals induced by nasal and mouth breathing have been a subject of interest in understanding how breathing patterns can influence brain activity and cognitive functions. Studies have shown that nasal breathing can significantly affect brain oscillations and connectivity, particularly in regions associated with cognitive processing and emotional regulation. For instance, nasal respiration has been found to entrain human limbic oscillations, which are crucial for cognitive functions such as memory and emotional processing. This entrainment is particularly evident in the piriform cortex, amygdala, and hippocampus, where oscillatory power peaks during inspiration [27]. In contrast, mouth breathing has been associated with different patterns of brain activity. Research indicates that mouth breathing can lead to reduced oxygen supply to the brain, which may affect cognitive performance and alter EEG patterns. For example, mouth breathing during cognitive tasks has been shown to result in lower theta and alpha power, which are critical for attention and working memory processes [28]. Additionally, mouth breathing has been linked to changes in functional connectivity, with more left lateralization observed in brain networks compared to nasal breathing, which tends to maintain a more symmetrical pattern [29]. Further studies have explored the impact of breathing patterns on EEG signatures during specific tasks. For instance, during a working memory task, mouth breathing was found to decrease the power of alpha and beta waves, which are associated with cognitive engagement and alertness, compared to nasal breathing [30]. This suggests that nasal breathing may support better cognitive performance by maintaining optimal brain activity patterns. Moreover, the modulation of EEG activity by breathing patterns has implications for understanding the neural mechanisms underlying cognitive and emotional functions. Nasal breathing has been shown to enhance the power and connectivity of the default mode network (DMN), a critical brain network involved in self‐referential thinking and emotional processing [31]. This enhancement is particularly pronounced in higher frequency bands, such as gamma, which are essential for complex cognitive functions. In summary, the differences in EEG signals induced by nasal and mouth breathing highlight the importance of breathing patterns in modulating brain activity and cognitive functions. Nasal breathing appears to support more favorable EEG patterns for cognitive and emotional processing, while mouth breathing may disrupt these patterns, potentially leading to cognitive deficits. These findings underscore the need for further research to explore the potential therapeutic applications of breathing techniques in enhancing cognitive and emotional well‐being.

### 4.2 Impact of Respiratory Flow Rate on EEG Signatures

Rapid breathing has been shown to significantly influence electroencephalogram (EEG) signal characteristics, distinguishing it from both normal and slow breathing patterns. This is particularly evident in the modulation of brain oscillations and the synchronization of neural networks. The impact of breathing on EEG signals is a subject of growing interest, as it provides insights into the interaction between respiratory patterns and brain function. One study demonstrated that different breathing rates, including rapid breathing, can alter the power of various EEG frequency bands, such as alpha and theta, which are associated with cognitive and emotional processing. Rapid breathing, in particular, has been linked to increased arousal and changes in cortical activity, which can be detected through EEG [32]. This is consistent with findings that suggest breathing patterns can modulate brain dynamics, influencing both the amplitude and phase of neuronal oscillations [33]. Furthermore, the synchronization between breathing and brain activity is not limited to changes in frequency power but also involves the coupling of respiratory cycles with neuronal oscillations. This coupling is more pronounced during rapid breathing, which can lead to enhanced coherence between different brain regions, as observed in studies examining the interaction between respiratory and cortical rhythms [27]. Such interactions highlight the role of breathing as a fundamental rhythm that can entrain brain activity, affecting cognitive and emotional states [22]. In addition to these effects, rapid breathing has been shown to influence heart rate variability (HRV) and autonomic regulation, which are closely linked to EEG signal characteristics. Studies have found that rapid breathing can decrease the high‐frequency component of HRV, reflecting a shift in autonomic balance that is mirrored in EEG changes [34]. This suggests that the physiological effects of rapid breathing extend beyond the respiratory system, impacting overall neural and autonomic function. Moreover, the impact of breathing on EEG signals is not only a matter of frequency modulation but also involves changes in the connectivity and functional organization of brain networks. Rapid breathing can enhance the connectivity between specific brain regions, potentially facilitating the integration of sensory and cognitive processes [35]. This is particularly relevant in the context of anxiety and stress, where rapid breathing is often observed and can exacerbate neural dysregulation [36]. Overall, the distinct EEG signal characteristics induced by rapid breathing compared to normal and slow breathing underscore the complex interplay between respiratory patterns and brain function. These findings have important implications for understanding the neural mechanisms underlying various physiological and psychological states and highlight the potential of breathing interventions in modulating brain activity and improving mental health.

### 4.3 Model Architecture Advantages and Performance Comparison

From the perspective of model architecture, the ResNet‐Swin fusion design shows breakthrough advantages. While multiscale convolution (16×/8×/4×) accurately captures the characteristics of local respiratory motion, the 7 × 7 window attention mechanism successfully models the dynamics of the whole brain network. This synergy solves the bottleneck of insufficient modeling of local details or global context by traditional methods and achieves more spatiotemporal coverage. The improvement of model robustness is manifested in the combination of cosine annealing scheduling and AdamW optimization strategy, achieving a faster convergence speed than conventional methods. The mechanism lies in that the adaptive learning rate adjustment suppresses weight oscillations. When combined with biometric alternatives (Table 3), our method shows that the accuracy of our method has increased by 4.17% compared with the motor image paradigm (98.3% compared with 94.13% of 64‐channel electroencephalogram) [14], and the accuracy of our method has increased by 19.0% compared with visual and audio stimuli evoked potentials (98.3% compared with 79.3%) [37]. The accuracy of our method has increased by 12.8% compared with face figure stimuli evoked potentials (98.3% compared with 85.5%) [38]. The accuracy of our method has increased by 15.2% compared with P300‐evoked response in EEG (98.3% compared with 83.1%) [18]. This advantage stems from the utilization of internal physiological rhythms rather than responses to external stimuli.

**TABLE 3:** Comparison of personal identification based on EEG.

Ref	Volunteer	Channel	Methods	Accuracy
[14]	109	64	Motor image paradigm	94.13%
[37]	5	14	Visual and audio stimuli	79.73%
[38]	10	18	Face figure stimuli	85.5%
[18]	4	8	P300‐evoked response in EEG	83.1%
This work	13	64	Respiratory‐evoked EEG	**98.3%**

The inherent fusion of high precision, noninvasive, and breath‐triggered characteristics provides a convincing opportunity for the clinical integration of respiratory medicine. Traditional methods of identity verification often fail in intensive care environments (e.g., intubated patients cannot provide fingerprints, delirium impairs password recall). Our approach offers a solution that integrates continuous, inconspicuous patient identity verification directly into respiratory monitoring, taking an important step towards safer and more patient‐centered clinical systems.

### 4.4 Clinical Translation and Implications

Although the respiratory–EEG biometric paradigm proposed in this study demonstrates significant technical advantages (98.3% accuracy), its clinical translation still requires addressing issues such as clinical workflow compatibility, artificial intelligence technical bottlenecks, and ethical and governance frameworks. Clinical workflow compatibility is crucial for the integrated system. The current 64‐channel EEG system faces operational complexities in ICU or home respiratory therapy scenarios (e.g., time‐consuming electrode placement and limitations in device portability). Future efforts should focus on developing medical‐grade wearable devices (e.g., respiratory masks integrated with EEG sensors) to achieve hardware coupling with ventilators and polysomnography devices. While current artificial intelligence models exhibit technical strengths in healthy populations, their performance in patients with respiratory diseases (e.g., abnormal breathing patterns in COPD) remains unvalidated. There is an urgent need to establish cross‐center pathological EEG databases and explore transfer learning frameworks to adapt to individualized respiratory variations (e.g., wheezing characteristics during acute asthma attacks). Potential ethical issues may arise, such as the irrevocability of EEG biometric features—once leaked, they could permanently compromise identity security. The EEG biometric identification system based on rapid nasal respiration triggering proposed in this study holds significant practical managerial significance, particularly in identity authentication scenarios within clinical environments. Firstly, the system addresses the limitations of traditional biometric methods in intensive care settings—intubated patients cannot provide fingerprints, while delirious states may impair password recall. Our respiration‐EEG coupled authentication method can be directly integrated into existing respiratory monitoring devices, such as home NIV therapy equipment, providing continuous and noninvasive identity verification solutions for patients with COPD/OSA. Secondly, this technology can be applied in polysomnography scenarios to enable automatic identity verification during sleep studies, avoiding disruptions to sleep states caused by traditional manual verification. Thirdly, in monitoring devices for opioid‐induced respiratory depression, the system can serve as a tamper‐proof security lock, ensuring that only authorized medical personnel can adjust parameter settings. From a medical management perspective, the core advantage of this technology lies in its dual integration of “treatment monitoring and security authentication,” which not only reduces identity management costs in clinical environments but also enables simultaneous patient status warnings through abnormal respiration pattern detection.

### 4.5 Open Research Questions and Future Directions

This study establishes a promising paradigm for respiratory‐evoked EEG biometrics, yet it inevitably raises several open research questions (ORQs) that chart the course for future investigation. These questions span sociotechnical integration, AI model development, and ethical governance. Addressing these ORQs will be crucial for translating this proof‐of‐concept into a secure, effective, and ethically sound clinical tool.

ORQ1: Sociotechnical and Clinical Workflow Integration.

How can we design seamless human–device interfaces that integrate this technology into complex clinical workflows without increasing the cognitive load on healthcare professionals?

The current reliance on a 64‐channel EEG system presents challenges in terms of setup time and portability in fast‐paced environments like intensive care units (ICUs). Future research must prioritize the development of miniaturized, medical‐grade wearable devices. A key direction is the creation of smart respiratory masks or ventilator interfaces with embedded, dry EEG electrodes. This hardware coupling would transform the biometric system from a standalone authenticator into an integral, unobtrusive component of therapeutic equipment, enabling continuous authentication during routine respiratory monitoring for conditions like COPD or sleep apnea.

ORQ2: AI Generalizability and Technical Bottlenecks.

Can AI models trained on data from healthy populations maintain high accuracy when applied to patients with pathological respiratory patterns or neurological conditions? The performance of our model in diseased states remains unvalidated. A critical future direction is the establishment of large‐scale, multicenter EEG databases encompassing diverse pathological conditions (e.g., asthma, COPD, and neurological disorders). Subsequently, exploring advanced transfer learning and domain adaptation frameworks will be essential to ensure model robustness across individual physiological variations. Furthermore, while our fusion model shows high accuracy, future work should investigate the interpretability of the model's decisions—understanding which spatiotemporal features of the respiratory–EEG signal are most discriminative could enhance clinical trust and provide insights into neurophysiological differences.

ORQ3: Ethical and Governance Frameworks.

What ethical guidelines and data governance structures are necessary to manage the unique risks associated with irrevocable biometric identifiers like brainwaves? Unlike a password, an EEG biometric trait is inherently linked to an individual and, if compromised, cannot be revoked or reissued. This irrevocability poses a significant security and privacy challenge. Future research must extend beyond technical accuracy to address these risks. This includes developing secure encryption methods for EEG templates stored on medical devices or in the cloud and establishing strict, transparent protocols for data ownership, consent, and access control. Proactive ethical analysis is needed to create governance frameworks that prevent misuse and ensure that the deployment of such systems prioritizes patient autonomy and privacy, particularly in vulnerable clinical populations.

### 4.6 Study Limitations

Of course, there are also some shortcomings in our study. We used 64 channels of EEG signals, which are somewhat redundant, especially to increase the model training time.

## 5 Conclusions

In this study, we developed a 64‐channel wireless EEG acquisition system and an event synchronizer. Based on this technology, we developed and implemented an experimental paradigm for EEG generated by various breathing patterns, as well as an EEG identity identification method based on Resnet and Swin transformer. The results demonstrate that using the ResNet & Swin Transformer model, the accuracy of nasal fast breathing is 98.3%. These mean that it is feasible to identify an individual based on rapid breathing‐induced EEG signals.

## Author Contributions

All authors have participated in this study and consent to publish this article in the Journal. The contribution list is shown in the following form: Guarantor of integrity of entire study: Cai Chen, Dedong Ma. Study concept: Cai Chen, Ningling Zhang, Fengxia Wu. Study design: Cai Chen, Xianghong Kong. Literature research: Cai Chen, Ningling Zhang, Danyang Lv, Xiangwei Meng. Data acquisition: Cai Chen, Ningling Zhang, Danyang Lv. Data analysis: Cai Chen. Manuscript preparation: Cai Chen, Xianghong Kong. Manuscript editing: Dedong Ma, Ningling Zhang. Manuscript revision/review: Cai Chen, Fengxia Wu, Dedong Ma.

## Funding

Special fund for the Key R&D Program of Shandong Province (2023CXPT039), Shandong Provincial Natural Science Foundation (ZR2021QH290), Mount Taishan Industrial Leading Talent Project, Shandong Province Key R&D Program of Shandong Province (2023CXGC010508), and Key Laboratory Project of Shandong Province (PKL2024C27).

## Ethics Statement

The study was conducted in accordance with the Declaration of Helsinki and approved by the Institutional Review Board (or Ethics Committee) of Ethics Committee of School of Nursing and Rehabilitation, Shandong University (2023‐R‐121).

## Conflicts of Interest

The authors declare no conflicts of interest.

## Data Availability

Data can be made available from the corresponding author.
